# Tailored Music Listening to Improve Sleep in Older Adults With Dementia: A Randomized Clinical Trial

**DOI:** 10.1093/geroni/igab046.901

**Published:** 2021-12-17

**Authors:** Darina Petrovsky, Nalaka Gooneratne, Joke Bradt, Miranda Varrasse McPhillips, Ime Etokebe, Laura Gitlin, Nancy Hodgson

**Affiliations:** 1 Rutgers University, Philadelphia, Pennsylvania, United States; 2 University of Pennsylvania School of Medicine, Philadelphia, Pennsylvania, United States; 3 Drexel University, Phialdelphia, Pennsylvania, United States; 4 University of Pennsylvania, Philadelphia, Pennsylvania, United States; 5 Drexel University, College of Nursing and Health Professions, Drexel University, Pennsylvania, United States; 6 University of Pennsylvania, School of Nursing, philadelphia, Pennsylvania, United States

## Abstract

Sleep disturbances in persons living with dementia (PLWD) contribute to reduced well-being. Music has shown promise to improve sleep among older adults, but there is limited evidence of music interventions improving sleep specifically in PLWD. The purpose of this wait-list RCT was to examine the i) feasibility; ii) acceptability and iii) preliminary efficacy of tailored music listening intervention in community-dwelling PLWD and their caregivers (dyads). Thirty consented dyads out of 33 (91%) completed the RCT. Tailored music for sleep was feasible based on screening (26%), enrollment (89%), and recruitment (3 dyads/month) rates. The intervention was found acceptable, as evidenced by post-intervention interviews. Compared to controls, PLWD in the intervention group reported greater global sleep quality improvement post-intervention (PSQI mean change -0.08 vs -1.65; p=0.06). The results from this feasibility RCT have informed the development of a music mobile application that will be tested in a future clinical trial.

